# Loss of PTEN expression is associated with colorectal cancer liver metastasis and poor patient survival

**DOI:** 10.1186/1471-230X-8-56

**Published:** 2008-11-26

**Authors:** Hirozumi Sawai, Akira Yasuda, Nobuo Ochi, Jiachi Ma, Yoichi Matsuo, Takehiro Wakasugi, Hiroki Takahashi, Hitoshi Funahashi, Mikinori Sato, Hiromitsu Takeyama

**Affiliations:** 1 Department of Gastroenterological Surgery, Nagoya City University Graduate School of Medical Sciences, Nagoya 4678601, Japan

## Abstract

**Background:**

The tumour suppressor phosphatase and tensin homolog (PTEN) is an important negative regulator of cell-survival signaling. To evaluate the correlation between PTEN expression and clinicopathological characteristics of colorectal cancer patients with and without liver metastases, we investigated PTEN expression in primary colorectal cancer and colorectal cancer liver metastases.

**Methods:**

Sixty-nine pairs of primary colorectal cancer and corresponding liver metastasis specimens were analyzed immunohistochemically, and the correlation between immunohistochemical findings and clinicopathological factors was investigated. Seventy primary colorectal cancer specimens from patients without liver metastases were used as controls.

**Results:**

PTEN was strongly expressed in 44 (62.9%) colorectal cancer specimens from patients without liver metastases. In contrast, PTEN was weakly expressed in 52 (75.4%) primary colorectal cancer specimens from patients with liver metastases, and was absent in liver metastases. Weak PTEN expression in colorectal cancer tissues was significantly associated with advanced TNM stage (*p *< 0.01) and lymph node metastasis (*p *< 0.05). PTEN expression was significantly stronger in primary colorectal cancer specimens from patients without liver metastases. Furthermore, among colorectal cancer patients with liver metastases, the 5-year survival rate was significantly higher in patients with positive PTEN expression compared to those with negative PTEN expression (*p *= 0.012).

**Conclusion:**

Our results suggest that loss of PTEN expression is involved with colorectal cancer aggressive capacity and that diagnostic evaluation of PTEN expression may provide valuable prognostic information to aid treatment strategies for colorectal cancer patients.

## Background

Colorectal cancer is the fourth most common malignancy and the second leading cause of cancer death in Western countries, with liver metastases being the primary cause of death in most patients [1]. Despite improved cancer screening methods and adjuvant treatment regimens for primary colorectal cancer, synchronous and metachronous liver metastases remain a critical problem [2,3]. Approximately 40% of patients who develop metastatic disease have tumour(s) confined to the liver, which has driven interest in regional therapies that target the liver [4].

An essential process for promotion of metastasis is cancer cell invasion, which is controlled by several biological factors, most notably tumour proliferation and invasion. The tumour suppressor phosphatase and tensin homolog (PTEN) is a critical negative regulator of the cell-survival signaling pathway initiated by phosphatidylinositol 3-kinase (PI3K) [5-9]. The PI3K-PTEN pathway promotes cell survival and proliferation, increases in cell size, and chemoresistance. Each of these biological outcomes results from the interaction of this pathway with other signaling networks. Further, loss of PTEN expression has been reported to be strongly associated with aggressive tumour features [10-13].

Controlling liver metastasis is considered essential in the treatment of colorectal cancer. It is therefore reasonable to expect that evaluation of PTEN expression levels in primary colorectal cancer and in liver metastases may provide information that will allow better prediction of therapy benefit. The aims of this study were to investigate PTEN expression in primary colorectal cancer and in colorectal cancer liver metastases and to evaluate the correlation between PTEN expression and clinicopathological characteristics of colorectal cancer patients with and without liver metastases. Our results suggest that loss of PTEN expression is associated with the aggressive capacity of colorectal cancer and that understanding the biologic mechanisms responsible for regulation of PTEN expression may enable better translational treatment of colorectal cancer patients.

## Methods

### Patients and tissue specimens

Sixty-nine pairs of primary colorectal cancer and corresponding liver metastasis specimens were analyzed in this study. Seventy primary colorectal cancer specimens from patients without liver metastasis were used as controls. All tissues were obtained in the Department of Gastroenterological Surgery, Nagoya City University Hospital with informed consent from patients or their relatives. Tissue samples were fixed in 10% formalin and subsequently embedded in paraffin.

### Immunohistochemistry

Resected colorectal cancer and paired liver metastasis tissue specimens were sliced into 3.5-μm-thick sections and deparaffinized in five min. After blocking nonspecific binding with 10% bovine serum (Wako, Osaka, Japan), sections were incubated with anti-PTEN antibodies (clone 28H6; Santa Cruz Biotechnology, Santa Cruz, CA, USA) at 1:300 dilution for 60 min at room temperature. Immunohistochemical staining was performed using the peroxidase-based EnVision™ kit (DakoCytomation, Copenhagen, Denmark) according to the manufacturer's instructions. Negative control sections were prepared using normal mouse IgG instead of primary antibody.

### Immunohistochemical evaluation

Two observers (H.T^a^. and H.S.) independently evaluated the immunohistochemical staining results. The concordance ratio was >90%. Differences of opinion were resolved by reaching a consensus with the assistance of a third evaluator (H.T^b^.). The intensity of tissue staining was graded semiquantitatively on a 4-point scale (-, +, ++, and +++). Likewise, the proportion of cells stained was assessed on a 4-point scale (1, 0–15%; 2, 15–50%; 3, 50–85%; 4, 85–100% cells stained). Tissues were classified into strongly staining (Group S) and weakly staining (Group W) groups according to the intensity and proportion of immunohistochemical staining. Immunohistochemical staining of intensity more than +++ or a staining area > 3 was classified as Group S, the intensity less than ++ and a staining area < 2 was classified as Group W.

### Statistical analysis

The Mann-Whitney *U *test was used to compare clinicopathological and immunohistochemical characteristics. Differences between Kaplan-Meier survival curves based on PTEN expression were tested with the Wilcoxon test. Statistical significance was indicated by *p *< 0.05 or 0.01. Data are presented as mean ± standard deviation (s.d.).

## Results

### Clinicopathological findings of patients with colorectal cancer with liver metastasis

Sixty-nine pairs of primary colorectal cancer and corresponding liver metastasis specimens were evaluated (Table 1). The mean age of all patients was 60.7 ± 9.9 years. pT, pN, and pM categories were determined according to the TNM classification [14]. The M category was determined from preoperative imaging findings, intraoperative findings, and postoperative imaging findings. A total of 23 patients had synchronous liver metastases and 46 had metachronous metastases; 38 patients had solitary liver metastases and 31 patients had multiple (≥ 2) metastases. Some patients received postoperative treatment; however, there was no difference in outcome between the various treatment modalities. The median follow-up time for patients at the time of survival analysis was 35.5 months after primary surgery. The actuarial 5-year survival rate of patients with colorectal cancer with liver metastases was 34.3%.

**Table 1:** Comparison of clinicopathological findings by existence of liver metastases

		**Patients with liver metastases n = 69 (cases)**	**Patients without liver metastases n = 70 (cases)**	***p *value**
Gender	M/F	40/29	38/32	N.S.
Age	Years	60.7 ± 9.9	62.5 ± 10.1	N.S.
TNM stage	I/II/III/IV	0/12/23/34	11/38/21/0	< 0.01^a^
Tumour site	C/R	45/24	49/21	N.S.
LN mets	Yes/No	41/28	31/39	N.S.
Differentiation	w/mod/por	37/30/2	21/33/16	N.S.
Liver mets	Sync/Meta	23/46	N.A.	N.A.
No. of liver mets	Solo/Multi	38/31	N.A.	N.A.
PTEN expression	S/W	17/52	44/26	< 0.01^a^
Median follow-up	Months	35.5 ± 27.4	37.2 ± 39.9	N.S.
5-year survival	(%)	34.3	79.2	< 0.01^b^

LN, lymph node; mets, metastasis; M, male; F, female; C, colon; R, rectum; w, well differentiated adenocarcinoma; mod, moderately differentiated adenocarcinoma; por, poorly differentiated adenocarcinoma; Sync, synchronous metastasis; Meta, metachronous metastasis; Solo, solitary metastasis; Multi, multiple metastases; S, strong; W, weak; N.A., not applicable; N.S., not significant. The ^a ^*p*-values were obtained using the Mann-Whiteny *U *test. The ^b ^*p*-values were obtained using Kaplan-Meier survival curves based on PTEN expression tested with the Wilcoxon test. Values of age are given mean ± s.d. N.S., not significant.

### Immunohistochemical localization of PTEN in colorectal cancer and liver metastasis specimens

**Figure 1:**
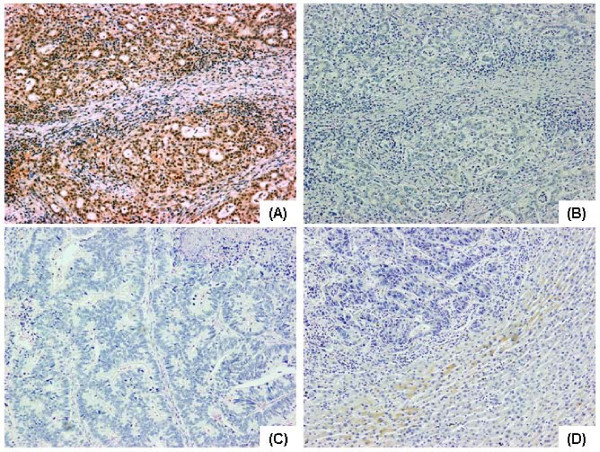
**Expression of PTEN in colon cancer specimens**. Immunohistochemistry was performed using a monoclonal anti-PTEN antibody (A, C) or negative control antibody (B) on a section sequential to that used in (A). (A) Strong PTEN expression in a representative moderately-differentiated adenocarcinoma specimen from colon cancer patients without associated liver metastasis (×100). (C) Expression of PTEN is not observed in a representative moderately-differentiated adenocarcinoma specimen from colon cancer patients with liver metastasis (×100). (D) Expression of PTEN is not observed in a specimen from metastatic liver tumor, in contrast, PTEN expression is observed in normal liver tissue (×100).

**Figure 2:**
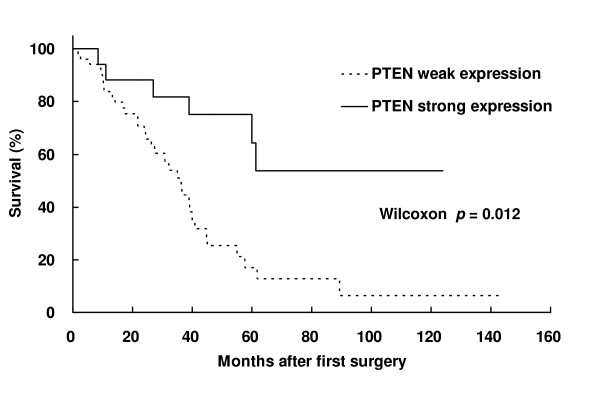
**Kaplan-Meier survival curves for colorectal cancer patients with liver metastases**. A comparison of survival curves between cases with (thick line) and without (broken line) strong PTEN expression.

PTEN expression was evaluated in primary colorectal cancer and corresponding liver metastasis specimens. PTEN was strongly expressed in 44 (62.9%) colorectal cancer specimens from patients without liver metastases (control, n = 70). In contrast, weak PTEN expression was observed in 52 (75.4%) primary colorectal cancer specimens from patients with liver metastases, and PTEN expression was absent in liver metastasis cancer specimens (Figure 1A–D). The marked nuclear staining of PTEN and slight cytoplasmic staining were observed. Weak PTEN expression was significantly associated with advanced TNM stage and lymph node metastasis (Table 2). The actuarial 5-year survival rate of patients with colorectal cancer with liver metastases and positive PTEN expression was 64.4%. In contrast, the actuarial 5-year survival rate of patients with colorectal cancer with liver metastases and negative PTEN expression was 12.7% (Figure 2). In addition, among colorectal cancer patients with liver metastases, there was a significant difference in 5-year survival rate between patients with positive PTEN expression and those with negative PTEN expression (*p *= 0.012).

**Table 2:** Comparison of clinicopathological findings by PTEN expression

		**PTEN expression**		
		**Group S (n = 17)**	**Group W (n = 52)**	***p *value**
Gender	M/F	10/7	30/22	N.S.
Age	Year	60.3 ± 9.7	60.9 ± 10.1	N.S.
TNM stage	I/II/III/IV	0/6/7/4	0/6/16/30	< 0.01^a^
Tumour site	C/R	11/6	34/18	N.S.
LN mets	Yes/No	8/9	39/13	< 0.05^a^
Differentiation	w/mod/por	10/7/0	27/23/2	N.S.
Liver mets	Sync/Meta	4/13	19/33	N.S.
No. of liver mets	Solo/Multi	9/8	29/23	N.S.
5-year survival	(%)	64.4	12.7	< 0.05^b^

LN, lymph node; mets, metastasis; M, male; F, female; C, colon; R, rectum; w, well differentiated adenocarcinoma; mod, moderately differentiated adenocarcinoma; por, poorly differentiated adenocarcinoma; Sync, synchronous metastasis; Meta, metachronous metastasis, Solo, solitary metastasis; Multi, multiple metastases; N.S., not significant. The ^a^*p*-values were obtained using the Mann-Whiteny *U *test. The ^b^*p*-values were obtained using Kaplan-Meier survival curves based on PTEN expression tested with the Wilcoxon test. Values of age are given mean ± s.d. N.S., not significant.

## Discussion

PTEN is a potent tumour suppressor gene that is frequently mutated in a large number of human cancers, including brain, endometrial, prostate, and kidney cancer. PTEN downregulates the activity of the lipid second messenger phosphoinositol-3,4,5-triphosphate (PIP3) through dephosphorylation, thereby negatively regulating PI3K-triggered signaling. The PI3K/Akt pathway is an important driver of cell proliferation and survival, most notably in cells responding to growth factor receptor engagement. Survival signals such as growth factors, cytokines, and hormones are known to activate PI3K [15]. Subsequently, PI3K activates Akt/PKB, which interferes with cellular apoptotic machinery [16]. Activated Akt/PKB mediates cell survival via the regulation of numerous proteins involved in apoptosis, such as the transcription factor NF-κB [17]. Akt-triggered survival signaling is suppressed by PTEN antagonization of PI3K activity.

PI3K and Akt are overexpressed in a variety of cancers [18,19], while PTEN is frequently deleted in advanced tumours [20,21]. These alterations lead to constitutively active survival signaling that enhances the insensitivity of tumour cells to apoptosis induction. Via PI3K antagonism, PTEN functions as a tumour suppressor. Thus, the PI3K-PTEN signaling network is crucial to proper regulation of cell survival. By reducing PI3K activity, PTEN inhibits the recruitment of Akt to the plasma membrane and prevents its activation. PTEN function is regulated by post-translational modification, cellular localization, and redox modulation of its active-site cysteine [22,23]. When PTEN is deleted, mutated, or otherwise inactivated, activation of PI3K effectors – particularly the key survival kinase Akt/PKB – can occur in the absence of any exogenous stimuli, allowing tumourigenesis to be initiated.

The molecular mechanisms by which PTEN controls stem cell behavior are currently unknown. Previously, PTEN loss has been reported to lead to an increase in self-renewal and proliferation in neural stem cells [24], at least in part, by modulating the G_0 _to G_1 _cell cycle transition, a critical step for stem cell self-renewal and differentiation [25]. Studies of mouse embryonic fibroblasts and B lymphocytes lacking the PTEN gene have demonstrated that these cells migrate faster than their wild-type counterparts in culture, indicating a physiological role for PTEN in the suppression of cell motility [26,27]. Re-introduction of PTEN into mammalian cells lacking the enzyme inhibits the motility of several cell lineages, including mouse embryo fibroblasts and tumour-derived cells of glial, prostate, and T cell origin [25,27-30]; however, the majority of these studies have not addressed the biologic mechanism of PTEN action.

Numerous tumour types, including sporadic tumours and those that arise in association with a cancer predisposition syndrome, demonstrate either absent or altered PTEN expression [31]. Recent reports demonstrated the relationship between PTEN expression and the efficacy of cetuximab, a chimeric IgG1 monoclonal antibody that targets the extracellular domain of epidermal growth factor receptor (EGFR) [32-34]. Frattini *et al *reported the loss of PTEN expression is observed in 40 % of primary colorectal cancer patients with synchronous or metachronous metastatic lesion and the loss of PTEN protein expression is associated with nonresponsiveness to cetuximab [32]. They also demonstrated a trend toward PTEN protein reduction in mucinous colorectal cancers, compared with well-differentiated tumors. In this study, we investigated PTEN expression in primary colorectal cancer and in colorectal cancer liver metastases and evaluated the correlation between PTEN expression and clinicopathological characteristics of colorectal cancer patients with and without liver metastases. In our immunohistochemical analysis, strong PTEN expression was observed in 62.9% of primary colorectal cancer specimens obtained from patients without liver metastases, while weak PTEN expression was observed in 75.4% of colorectal cancer specimens obtained from patients with liver metastases. In addition, among colorectal cancer patients with liver metastases, we found that weak PTEN expression was significantly associated with advanced TNM stage and lymph node metastasis. Furthermore, among colorectal cancer patients with liver metastases, a significant difference in 5-year survival rate was observed between patients with positive PTEN expression and those with negative PTEN expression. This is the first report to demonstrate an association between poor prognosis and PTEN expression in colorectal cancer patients with liver metastases.

## Conclusion

In this study, we found that weak PTEN expression is significantly associated with advanced TNM stage and lymph node metastasis. This is the first report to demonstrate a significant difference in 5-year survival rate between patients with positive PTEN expression and those with negative PTEN expression among colorectal cancer patients with liver metastases. These observations suggest that diagnostic evaluation of PTEN expression might provide valuable prognostic information to aid treatment strategies for colorectal cancer patients.

## List of abbreviations

PTEN: phosphatase and tensin homolog; PI3K: phosphatidylinositol 3-kinase; PIP3: phosphoinositol-3,4,5-triphosphate; EGFR: epidermal growth factor receptor.

## Competing interests

The authors declare that they have no competing interests.

## Authors' contributions

HS, AY, NO, HTakahashi, MS, and HTakeyama conducted clinical examinations and surgery. HS, TW, YM, HF, and JM performed pathological analysis. HS, HTakahashi, and AY participated in the design of the study. HS and HTakeyamaconceived of the study and participated in its design and coordination. All authors read and approved the final manuscript.

## Pre-publication history

The pre-publication history for this paper can be accessed here:


